# The experience of good mentoring focused on overcoming turnover intention among millennial nurses

**DOI:** 10.3389/fmed.2024.1288829

**Published:** 2024-02-07

**Authors:** Seo-Yeon Jung, Jung-Hee Kim

**Affiliations:** College of Nursing, The Catholic University of Korea, Seoul, Republic of Korea

**Keywords:** mentee, mentors, mentorship, focus groups, nurses, qualitative research

## Abstract

**Introduction:**

Millennials are emerging as a prominent demographic in the nursing workforce. It is necessary to create an environment that harmonizes the advantages of each generation in a nursing workforce in which various generations coexist. As the importance of mentoring programs for millennial nurses grows, it is believed that the effectiveness of mentoring to support millennial nurses can be enhanced by identifying the attributes of good mentors as perceived by nurses. This study aimed to explore the attributes of good mentors according to millennial nurses in the nursing workforce with a focus on overcoming turnover intention problems.

**Methods:**

Four focus group interviews were conducted to collect data, which were subsequently analyzed using Braun and Clarke’s thematic analysis method. A descriptive qualitative design involving 22 millennial nurses employed at a tertiary hospital, Hospital A, in Seoul, Republic of Korea was utilized.

**Results and discussion:**

Four themes emerged from the analysis: the concept of a significant others, the presence of a driving force to endure, the importance of a guide to a harmonious life, and the value of a partner for growth. The participants themselves identified these themes. To prevent turnover intention among millennial nurses, mentors should employ diverse strategies, and institutional supports are crucial. Furthermore, since it is unrealistic to expect all ideal mentor characteristics to be present in one person, mentor development education is also necessary. This information is valuable for designing mentor development programs and for establishing a solid framework for effective mentoring programs.

## 1 Introduction

As the baby boomer generation continues to retire, millennials are emerging as a prominent demographic in the nursing workforce (1). Individuals born between 1981 and 1996 are typically categorized as belonging to the millennial birth cohort (2). Millennial nurses experience a high turnover rate and resignations due to their increased desire for work-life balance. The shortage of skilled nurses resulting from the substantial turnover rate is expected to increase workloads and worsen workforce shortages (3) ultimately leading to global cost implications (4).

It is necessary to create an environment that harmonizes the advantages of each generation in a nursing workforce in which various generations coexist (5). According to prior research, as the values between mentors and mentees align, nurse satisfaction tends to improve, turnover rates decrease, and ultimately, patient outcomes are enhanced (6–8). Furthermore, mentorship facilitates the development of leadership skills among nurses within multi-generational nursing organizations (1).

Mentoring is characterized as a relationship-centered process between mentors and mentees. In general, among the qualities of an ideal mentor, personal aspects include an outgoing and interactive personality, kindness, justice, and altruism (selflessness). Professional aspects of good mentors include being cooperative, intellectual, and proficient (9). In other studies, active listening, support, consideration, honesty, constructive feedback, self-reflection ability, and open communication abilities were determined to be qualities of good mentors (10–12). Therefore, mentors should have the skills they need to perform their roles, responsibility, a desire to help others grow and develop, and expertise in their areas (13). For a nursing leader, a mentor should be knowledgeable, supportive, and willing to invest time. In addition, they should be generous, resourceful, willing to share their own networks, open to new ideas, and ready to take on risks (14).

As the importance of mentoring programs for millennial nurses grows (15), it is believed that the effectiveness of mentoring to support millennial nurses can be enhanced by identifying the attributes of good mentors as perceived by the nurses. Faculty working in academia have also emphasized the importance of mentoring because it is an effective approach to help foster leadership (14). Numerous studies have explored the characteristics of good mentors (10, 16, 17). However, no studies have identified the roles and relationships of mentors with clinical nurses in the millennial generation.

Existing studies have focused on factors influencing nurses’ turnover and resignation (18, 19) including cost issues (3), but have not considered the characteristics of their generations. Millennials value building rapport as well as communication with their superiors. They believe that sharing their opinions and ideas and presenting creative solutions are more beneficial to the work place (20). Millennials also generally tend to seek meaningful work with opportunities to learn and grow. Thus, they choose many nursing jobs for growth and career development (21).

Focus group interviews are part of a qualitative research method that allows researchers to explore participating groups’ knowledge, beliefs, perspectives, and attitudes through mutual dialogue and discussion among by asking specific questions about research themes (22). This is an accepted qualitative research method due to the collection of in-depth data and provision of more details of the theme under study (23).

Therefore, this study aimed to explore the experience of good mentoring according to millennial nurses in the nursing workforce with a focus on overcoming turnover intention problems. The finding of this study elucidate the attributes of good mentors as determined by millennial clinical nurses and provide a new perspective that considers their relationships with mentors to help overcome turnover intention and increase the number of millennial clinical nurses within the nursing workforce.

## 2 Materials and methods

### 2.1 Design

This study adopted a focus group interview to describe and explain the attributes of good mentors as perceived by millennial clinical nurses working at a tertiary hospital. The study followed the Consolidated Criteria for Reporting Qualitative Studies, which covers the reporting of studies using interviews and focus groups (24).

### 2.2 Participants

The study involved millennial clinical nurses at a tertiary hospital, Hospital A, in Seoul. The inclusion criteria were as follows: nurses who provided direct patient care in the clinical field for more than six months. The exclusion criteria were as follows: non-millennial clinical nurses, millennial nurse managers who did not participate in patient direct care, and those who had been on leave within the previous six months.

Participants were recruited by posting and promoting online notices to nurses at Hospital A, explaining the purpose and research method of the study through in-hospital mail, and recruiting nurses who voluntarily applied for participation in the study.

The number of focus group participants typically ranged from 6 to 12; in this study, up to 6 people were grouped for free discussion based on the theme. Considering the nursing work environment, we divided the participants into four groups.

### 2.3 Ethics

This study was approved by the Research Ethics Committee of a tertiary hospital, Hospital A (IRB number: 2020-1509). The participants were assured of confidentiality and informed that participation was voluntary and that they could withdraw from the study at any time. They were informed that the transcripts would not be used for any purpose other than research.

### 2.4 Data collection

Data were collected via focus group interviews conducted in April 2022. The focus group in this study included millennial clinical nurses (the oldest born in the 1980s and the youngest in the 2000s) who were classified into the following groups: nurses born between 1980 and 1989 and nurses born between 1990 and 1997. Each group was again divided into subgroups of internal medicine and surgery. The interviews were conducted through Zoom platform in their house rooms or meeting rooms in the hospital, which were quiet and separate places because face-to-face interviews were not possible due to the COVID-19 pandemic restrictions. During the interviews, all participants were allowed to use a pseudonym or freely turn the camera on or off. The burden of the interviews was minimized, and the participants were allowed to talk freely.

A typical focus group interview requires 45–90 min; in this study, each interview lasted approximately 100 min per group. The interviews were conducted after receiving the participants’ consent for participation and interview recording in Korean. During the interview, the researcher provided a warm, accepting environment that encouraged all group members to share their views and added comments and asked probing questions to clarify their experiences. Effort was made to ensure that the researcher’s thoughts or experiences did not influence the participants’ statements, thereby maintaining a sense of neutrality in the research process.

Each group interview was allowed to continue until saturation (25). The data for the study included the recorded contents of the focus group interviews, and the recorded data were used for analysis along with the researcher’s transcribed notes that were prepared during the interviews. Regarding the content of the group interviews, a questionnaire was created to solicit specific content related to the purpose of the study from everyday content, and the discussion was conducted in a “floating” manner (26). The questions included opening, transition, key, and ending questions (27). The main questions were as follows: “What does it mean to be a good mentor for the ‘millennial’ generation clinical nurse?”, “What kind of mentor-mentee relationships do you think you need?”, and “What kind of mentor-mentee relationships can prevent nurses from changing jobs and resigning?”

### 2.5 Data analysis

The transcribed data were analyzed using Braun and Clarke’s thematic analysis method (28). A thematic analysis is a valuable method for analyzing subjects’ perspectives; it is flexible and requires analysis of potential meanings rather than assessment of the frequency of appearance of words (29). The thematic analysis included six steps.

The first step was to repeatedly read the collected data to become familiar with them. The second step was the initial coding generation. The initial code was formed by systematically identifying the characteristics that were observed throughout the data. The third step was to collect the initial code written as a theme-finding step to identify themes that could contain and express the common content of the code and to combine the relevant codes. In the fourth step, the following factors were examined: similarities and differences, identifiability and correlation, amount of code, and consistency and clarity of the pattern. The fifth step was an analysis step to identify the characteristics of each theme that was named while clarifying its nature and definition. The last step was to write a report, and the results were accurately described while maintaining consistency and logic (28, 29).

Nowell et al. (30) have recommended that the credibility, transferability, dependability, and confirmability proposed by Lincoln and Guba (31) should be promoted to ensure the rigor of a subject-analysis study; this study was conducted according to this recommendation. The participants’ interviews were transcribed, and the transcription was checked for consistency with the recording. Three interviewees verified the derived results to confirm that the main contents were reflected. Themes and subthemes were examined by two nursing professors with experience in qualitative research and one who majored in education.

## 3 Results

Regarding the participants’ general characteristics, 10 and 12 nurses were born in the 1980s and 1990s, respectively, and all of them worked at a certified tertiary hospital in Seoul. Among them, 12 and 10 nurses worked in the internal medicine department and surgery department, respectively. All the participants were female, the average age was 31.6 years, the average nursing experience was 7.3 years, and 3 out of the 22 nurses responded that they had experience in mentoring programs.

A total of 587 meaningful statements were extracted from the participants’ experiences with good mentors, and 88 concepts and phrases were derived through open coding. These were integrated into 68 subcategories, which were structured into 9 categories, and 4 themes were derived: “a significant other” “a driving force to endure,” “a guide to a harmonious life,” and “a partner for growth” (Table 1).

**TABLE 1:** Summary of theme, categories, and sub-categories.

Theme	Categories	Subcategories
A significant other	Time and experience together	Sharing time and experience
		Understanding of mentees
		Intimate exchange
	Emotional connection	Empathy for mentee
		Love and trust in mentee
		Being reliable
		Being comfortable
		Empathetic communication
		Empathy
		Mentor’s authenticity
		Adviser
		Forming a deep bond
A driving force to endure	Breakthrough in an ordeal	Empathy and comfort for difficulties
		Problem-solving skills
		Consideration
		Social parents
		Being completely on my side
	Centering oneself	Making a feeling of belonging
		Being dependable
		Presenting a vision
		Support for professional skepticism
		Healing the inner wounds
		Responsibility
		Role as a self-help book
	Discovering the meaning and value of nursing	Adherence to nursing principles
		Patient-centered nursing
		Professional qualifications
		Finding the meaning of nursing
		Exploring the philosophy and nature of nursing
		Enhancing the professional spirit
		Making me realize the importance of nursing
		Making me discover my own nursing care
A guide to a harmonious life	Looking back at oneself	Breaking the narrow frame
		Broadening my horizons
		Self-reflection
		A wider understanding of others
		Insight
		A capacity to embrace
		Making me find my identity
		Filling in the gaps
	Ideal life	Interpersonal skills
		Ideal self
		A sense of security
		A positive attitude
		Role model
		Drawing a blueprint
		A balance between work and life
A partner for growth	A stepping stone to growth	Not to blame
		Motivation for growth
		Believing in a mentee’s potential
		Instilling a sense of challenge
		Developing self-reliance and giving courage and wisdom
		Helping mentee grow
		Identifying strengths and weaknesses
		Reinforcing the strength of a mentee
		Compensating for a mentee’s weaknesses
		A sense of purpose
		Giving directions
		Being a milestone
		A hopeful future
	Dreaming of being a future mentor	Improvement of the nursing environment
		Desire to be a good mentor
		Helping self-development
		Good influence
		Being an example for other nurses
		Leading
		A stimulant to the soul
		Prevention of turnover and resignation

### 3.1 Theme 1: a significant other

In the years a mentor and mentee spend together, a theme based on mutual understanding became apparent. The participants perceived mentors who had such a relationship as significant others. Communication with mentors and their advice was sincerely accepted in a relationship based on positive emotions about them, and mentees learned more about mentors and thought more about what they could learn from them in this type of relationship.

#### 3.1.1 Time and experience together

All the interviewees considered time and experience together as important factors. They described that good mentors were not made by external factors such as good academic background, outstanding careers and specifications, and long-term work experience but by their characteristics of having understanding and empathy for their mentees and having comfortable and intimate relationships. Time and experience together as well as the maintenance of a mentor-mentee relationship even if a situation of transferring departments occurred were considered important.

*“I think it is essential that the mentor knows me well—for example, my personality’s strengths, weaknesses, and tendencies. Thus, I believe time sharing each other’s experiences in the same ward affects becoming a mentor.” (80 Internal Medicine-5)*

#### 3.1.2 Emotional connection

They also thought that a good mentor was not a problem solver or advisor but a person who could have a comfortable and warm relationship that involved sharing feelings. With regard to advice on hard work, they felt that comfortable relationships should come first and that advice given in the context of such relationships was more authentic.

*“As a mentor, it is essential to know the mentee’s main problem; however, more importantly, I think a mentor and a mentee’s relationship should be one in which they can positively communicate and advise on such issues.” (80 Internal Medicine-5)*

### 3.2 Theme 2: a driving force to endure

This theme included millennial clinical nurses’ experiences of the transition period in the process of growing. The participants felt they could express their difficulties to and be comforted by mentors with whom they shared mutual trust. Their mentors were the driving forces for them to continue their work without leaving a complex clinical site. Through good mentoring, the participants could heal from bullying that they had undergone, and they did not wander; they learned through their mentors by using them as milestones, found the meaningfulness of nursing, and felt proud of their nursing jobs. Through this process, the participants felt that they could comprehend the essence of nursing anew and that they could find their own care philosophy.

#### 3.2.1 Breakthrough in an ordeal

Participants felt that working in intensive care nursing, responding to caregivers, and handling nursing responsibilities in conflicts with other professionals were challenging. They expressed that a good mentor is a person who helped them overcome various trials in a difficult clinical field, and that the role of a mentor is a social parent who fosters mentees in society.

*“When something difficult happens, I think of her first, and I think it will be solved if I talk to her; thus, I want to discuss it at any time. Looking at her makes me think that I want to be like her.” (80 Internal Medicine-4)*

*“When we are born, we have teachers called parents, and when we enter society, we think of mentors as the parents of society. Mentors seem to play a role in nurturing mentees so that they can grow in the community.” (80 Internal Medicine-6)*

#### 3.2.2 Centering oneself

In many cases, the participants thought of resigning because of troubled relationships with people such as colleagues and patient caregivers or because of physical and mental fatigue. However, the participants have not left the nursing field thus far because they have mentors who help them take center stage when challenges arise and keep them from wandering.

*“For mentees, a mentors’ inadvertent actions or words can significantly impact them. That is why I think that a sense of responsibility for mentees is the most important. When a mentee wanders too much, it is hard on the mentor. However, I believe it is vital for a mentee to have a mindset to try not to give up halfway.” (90 Surgery-2)*

#### 3.2.3 Discovering the meaning and value of nursing

The participants felt that they could reflect on the meaning and essence of nursing and learn the importance of patient-centered nursing by having a mentor who builds professional knowledge and practices patient-centered nursing.

*“For me, a good mentor was the driving force that allowed me to endure challenging clinical fields and the kind of person who enhanced my professional spirit as a nurse. A true mentor would make me proud of my nursing job and allow me to establish the reason for my existence by following them.” (90 Internal Medicine-2)*

### 3.3 Theme 3: a guide to a harmonious life

When the participants lost their identity as nurses, they could broaden their horizons with their mentors’ advice and have the opportunity to look back and reflect on themselves while being helped to break the narrow framework. In addition, mentors were the ones who served as a blueprint for the participants’ desired lifestyles, and the participants thought that a good mentor was a person who achieved the harmonious work-life balance that they valued and whom they wanted to resemble.

*“When I was a novice, I thought that a mentor worked well, immersed themselves, and achieved something. However, recently, I think that someone who works and lives in harmony is more like a mentor than someone who works too much—someone who can emulate something outside of work.”*

*(80 Internal Medicine-1)*

#### 3.3.1 Looking back at oneself

Participants experienced burnout while playing various roles in the hospital, and their identity as nurses was shaken, and they wandered. However, through mentors, they were able to break a narrow frame of mind and look back at themselves to remedy their shortcomings, and that these qualities of a mentor were not related to their age or career status.

*“I think a mentor should help me find my identity and support and help me when I feel professional skepticism. What am I doing? Who am I as a nurse now? When I think about it, I think the mentor holds a positive mental status and allows me to move forward.” (90 Internal Medicine-4)*

#### 3.3.2 Ideal life

Participants were able to find their identity as nurses through mentors and wanted to resemble them. In addition, they used their mentor’s lives as a blueprint to strengthen their future and their will to live such a life.

*“I draw a blueprint for my job and personal life by looking at my mentor, who is constantly growing. When I obtain advice, I hope to manage my life well; thus, I feel that a mentor is significant. Furthermore, as I am in the ward, I feel the limitations as I grow older; however, talking about this and that together relieves my frustration and strengthens my will by looking at her steps ahead.” (90 Internal Medicine-5)*

*“I want to obtain advice from someone like that and follow that path. That kind of person is a mentor, and it seems to change gradually.” (80 Internal Medicine-1)*

### 3.4 Theme 4: a partner for growth

Through good mentoring, the participants also wanted to become good mentors who could have a good influence on their mentees, and they felt that mentoring was about mentors and mentees growing together. The participants recognized that it was challenging but necessary for nurses to be good mentors and they dreamed of being good mentors who could compensate for their mentees’ shortcomings and affect their overall lives and work through their experiences.

#### 3.4.1 A stepping stone to growth

Good mentors played a role as stepping stones for the participants to grow. When thinking about resignation and job changes, mentor provided a direction and became a milestone in the future. Participants were able to look back on the past and evaluate the present and envision a hopeful future through good mentors, and they were still looking for mentors to help them grow in the present.

*“Give direction. What can I do with what I have built up here? Whenever I go through a hard time, I want to stop; however, watching my mentor plan for the future helps me gain new knowledge and broaden my perspective.” (90 Internal Medicine-3)*

*“Regardless of age, trust me, sharing the mentee’s concerns, and my words making their life better. It can have a good influence on work, interpersonal relationships, and other areas*… *I do not merely want to emulate it, but I think a mentor who gives spiritual stimulation is a true mentor, and I think clinical nurses need it. If possible, I would like to be a mentor like that.” (90 Surgery-2)*

#### 3.4.2 Dreaming of being a future mentor

Participants recognized that it was difficult but essential for nurses to be good mentors, and through their experience with their mentors, they dreamed of becoming good mentors who could compensate for their mentee’s shortcomings and affect their mentees’ lives as well as work.

*“As a colleague who has the same job, I would be happy and proud if I could help someone. I also want to be a mentor because I think it is a way for people who use me as a mentor and those who become mentees to develop together.” (90 Internal Medicine-5)*

## 4 Discussion

This study utilized a thematic analysis that applied focus group interviews to explore the attributes of good mentors as experienced by millennial nurses in nursing workforces with a focus on overcoming turnover intention problems. The results of this study could provide information about the roles of good mentors based on millennial nurses’ experiences in the nursing workforce and provide a new perspective that considers relationships with mentors as part of a strategy to prevent nurse shortage within the nursing workforce.

First, millennial clinical nurses valued their time and experience with their mentors. Time, space, and scheduling constraints have been described as the most common obstacles limitations for mentoring (32). As mentoring is based on an individual-to-person relationship (33, 34), common professional and personal interests and experiences during the time spent together are essential to developing a good relationship (35). This is consistent with research indicating that time for trust-building and studies are necessary to avoid perceiving mentor feedback as personal criticism (36, 37). Empathy was identified as an essential factor in the quality of relationships in youth mentoring (38), whereas it was also evaluated as a critical quality of mentors by millennial subjects The participants also emphasized the importance of communication: millennials want to meaningfully communicate with those who respect and defend themselves and support their professional goals and success (39).

In prior studies, the qualities of effective mentors have been discussed (10–14). However, this study found that effective mentors are not problem solvers but have the qualities to understand and support their mentees’ values. As mentoring includes voluntary and long-term relationships (25), developing personal characteristics and communication skills such as empathy and respect is essential for mentors and can be improved with education and organizational support.

Millennial nurses considered good mentors as “a driving force to endure.” In Korea, negative peer relationships, unfavorable work environments, excessive workload (26), and lack of support from nursing organizations (27), represented by the Korean nurse Taeoom (40), are known to be the main reasons for nurses leaving their work. Despite the high growth motivation among millennial nurses, they experience more stress and exhaustion than other generations, leading to a higher level of turnover (41). Through mentoring, mentors can help develop mentees’ self-concept and self-efficacy, thereby strengthening nurses’ motivation and confidence in their work as well as their organizational commitment (41, 42). In this study, participants felt that they could increase their self-efficacy, learn nursing skills, and find the meaning of nursing through good relationships with their mentors. Good mentoring could be one way to provide millennials with a sense of accomplishment for their roles and to retain them in their work (8). Nursing organizations should provide institutional support for mentoring programs such as organizational socialization to improve peer relations and preparation of communication channels to improve the work environment. In addition, it is necessary to develop leadership programs for millennial nurses that can support their strengths and weaknesses. This would enable the development of skilled nursing personnel, and systematic program evaluation is required for continuous improvement.

The participants considered a good mentor as “a guide to a harmonious life.” Millennials want to achieve new things, develop professional identity (43), and feel strong, inner job satisfaction (44). However, they are not motivated by the traditional system that seeks to control their personal lives (44); they value time balance for individual and social life (42) and define work-life balance as success that deserves promotion (41, 45). This means that they regard their jobs not as mere jobs but as a part of their lives in pursuing development (46). This is the most important difference with “baby boomer” generation. “Boomers” are usually “workaholics” and are willing to sacrifice their personal lives and interests for the sake of work (47). Therefore, millennials may experience conflict between their expectations for the roles of managers, seniors, and leaders and their desire to pursue work-life balance (48). Therefore, a mentor should have understanding and insight about the work values and engagement of different generations and be able to embrace generational differences. Mentor training that facilitates the integration of work- life balance is also necessary (49).

The participants regarded a good mentor as “a partner for growth.” The development of nursing professionalism is an essential factor in the growth of nurses, and mentoring is considered one of the crucial strategies for this; it can promote mentees’ knowledge by learning, obtaining advice, and sharing insightful thoughts from their mentors’ experiences (46). Mentoring positively impacts mentees; however, mentors can be equally rewarded (50). In addition, effective mentoring is an impressive experience related to interactions with mentors, which induces further mentoring (51). To prevent nurse turnover, mentors play a vital role in conducting comprehensive career assessments, offering professional advice, and providing education and training aligned with nurses’ goals. A mentor’s and mentee’s needs, expectations, and perceptions may be quite different (52). In this study, participants wanted a mentor as partner for growth. During the mentoring process, if the direction is not aligned as expected, the mentee should have the autonomy to terminate the relationship at any given time.

Based on the result of this study, nursing organizations should consider the mentee’s key concerns and values, ensure the goal of the mentoring program, match the mentee with a mentor who is qualified, and provide a regular meeting process for the formation of relationship between the mentor and mentee when develop mentoring programs.

There are several studies that have attempted to improve the adaptation and clinical skills of new nurses (53–55), but mentoring programs for millennials who are major skilled nurses have not been receiving attention. This study highlights the necessity for institutional support geared toward contributing to the advancement of nursing by instilling a sense of accomplishment in millennials, mitigating burnout, and enhancing their capabilities, and mentoring has been identified as a pivotal component of these efforts.

This study has the following significance: it can provide a new perspective that considers generational characteristics to help maintain and increase the number of millennial clinical nurses within the nursing workforce. Second, it can provide basic data for developing a mentor competency evaluation tool for millennial nurses. Mentor competency evaluations can help organizations offer feedback to mentors to improve their mentoring relationships and help mentees select the mentors they want (56). The present study will assist nurse educators in developing professional development to enhance mentors’ capacities. The themes of this study will also serve as a framework for developing successful mentoring programs for new nurses to successfully adapt to clinical work environments.

### 4.1 Limitations

This study has some limitations. Our findings do not represent all millennial nurses’ experiences because all the participants were female and because they voluntarily participated in the interviews. The study also exclusively focused on Korean nurses, and cultural differences may exist. Owing to subjective understanding, there may be errors and biases in the quality and quantity of nurses’ experiences; thus, the results should be interpreted carefully. Using webinar platform was possible to provide comfort to the participants, but the interview time was limited due to cost issues. Therefore, in future studies, it is necessary to study male nurses, who account for a minority of nursing personnel, and millennial nurses from other cultures and to involve larger samples to validate the findings of this study. Nevertheless, mentors’ attributes that can contribute to reducing millennial clinical nurses’ turnover intention and supporting their growth in the nursing workforce were identified. Information for the development of mentoring programs was also provided.

## 5 Conclusion

This study highlights the importance of exploring generational values in successful mentoring relationship. In the development of successful mentoring programs, nursing organizations should consider mentees’ key concerns and values and the goal of the mentoring program, select mentors who are qualified to understand and support their mentees’ concerns, and provide the time for the formation of relationships between mentors and mentees.

These efforts will enhance job satisfaction and support the growth of the millennial nursing workforce. Therefore, it will be crucial to consider generational values in mentor selection and program content when developing mentoring programs. Nursing organizations should engage in introspection and systematic evaluation to address challenges associated with the retention of millennial clinical nurses.

## Data availability statement

The raw data supporting the conclusions of this article will be made available by the authors, without undue reservation.

## Ethics statement

The studies involving humans were approved by the ASAN Medical Center (IRB number: 2020-1509). The studies were conducted in accordance with the local legislation and institutional requirements. The participants provided their written informed consent to participate in this study.

## Author contributions

S-YJ: Conceptualization, Investigation, Writing – original draft. J-HK: Conceptualization, Methodology, Supervision, Visualization, Writing – review and editing.
